# Various Blood Glucose Parameters that Indicate Hyperglycemia after Intravenous Thrombolysis in Acute Ischemic Stroke Could Predict Worse Outcome

**DOI:** 10.1371/journal.pone.0094364

**Published:** 2014-04-18

**Authors:** Deok-Sang Yoo, Jane Chang, Joon-Tae Kim, Min-Ji Choi, Jina Choi, Kang-Ho Choi, Man-Seok Park, Ki-Hyun Cho

**Affiliations:** Department of Neurology, Cerebrovascular Center, Chonnam National University Hospital, Gwangju, Korea; University of Glasgow, United Kingdom

## Abstract

**Background:**

Hyperglycemia is common after stroke, and it is well known to worsen its outcome. However, it is important to consider that blood glucose (BG) levels can undergo dynamic changes during the acute stage of ischemic stroke. We sought to investigate the clinical significance of various glucose parameters within first 24 hours in acute ischemic stroke (AIS). The study focused on hyperacute stage patients who underwent IVT and investigated which parameters of glucose demonstrated to be helpful for predicting outcome.

**Methods:**

This was a retrospective study of consecutive patients with AIS at a single stroke center. Patients were consecutively enrolled if they were treated with IV-tPA within 3 hours of symptom onset. BG was measured immediately upon arrival in ER, after IVT and every 6–8 hours during the first 24 hours after IVT. The various parameters of BG were the following: BG before IVT, BG after IVT, mean BG (mBG), maximal BG (max BG), standard deviation of BG (sdBG), and standard deviation of mean BG (sdmBG).

**Results:**

207 patients (127 men and 80 women) were included in this study. Seventy seven of 207 patients had favorable outcomes at 3 months. High BG after IVT, mBG and max BG were independently associated with mRS>2 at 3 months (adjusted by age, NIHSS, and atrial fibrillation). Several parameters of BG were also independently associated with early mortality within 3 months (BG after IVT, mBG, and max BG). BG after IVT and mBG over 180 mg/dL were independently associated with early mortality within 3 months.

**Conclusion:**

Serial measurements of BG might be a better predictor of clinical outcome in patients with AIS treated with IVT than single BG measurements before IVT. Therefore, these results suggest that variable parameters of BG could be important for the prediction of clinical outcome in AIS treated with IVT.

## Introduction

Hyperglycemia is common after stroke, and it is well known to worsen its outcome [1]–[3]. Even in patients treated with intravenous thrombolysis (IVT), a single glucose measurement of admission hyperglycemia has been found to be associated with poor outcome [4]. However, it is important to consider that glucose levels can undergo dynamic changes during the acute stage of ischemic stroke [5], in which these fluctuations may be correlated with stroke severity within the first 24 hours following onset [6]. A previous study suggested that hyperglycemia in acute stroke might primarily be an epiphenomenon to stroke severity [5]. The investigators hypothesized that because glucose levels may be associated with stroke severity, delayed glucose examinations would have negligible clinical significance. Although post-stroke hyperglycemia has not been proven definitively to be a causal factor for poor outcome after stroke, several mechanisms have been identified through which hyperglycemia could aggravate cerebral damage in ischemic stroke, including impaired recanalization and reperfusion injury [7], [8].

A recent study found that blood pressure variability rather than mean blood pressure was more associated with outcome in ischemic stroke [9]–[11]. Similarly, blood glucose variability was found to be an important factor in predicting mortality for critical illnesses like sepsis [12], [13]. While blood glucose will initially increase during the acute stage of stroke, there may be considerable fluctuations, and these fluctuations can contribute to outcome. Therefore, serial blood glucose measurements could better predict changes in glucose levels and hence aid in predicting outcome.

We sought to investigate the clinical significance of various glucose parameters in the acute stage of ischemic stroke. In order to eliminate the effect of stroke severity on glucose in delayed examinations, the study focused on hyperacute stage patients who underwent IVT, investigating which parameters of glucose demonstrated to be helpful for predicting outcome.

## Methods

### 1. Patients

This was a retrospective study of prospectively registered patients with acute ischemic stroke at our tertiary stroke center between January 2011 and July 2012. Patients were consecutively enrolled if they (1) had an acute ischemic stroke within 3 hours of symptom onset, (2) had a National Institute of Health Stroke Scale (NIHSS) score of ≥4, (3) had acute ischemic lesions on diffusion-weighted imaging (DWI), and (4) were treated with IV-tPA. We excluded patients (1) who had other etiologies, such as Moyamoya disease, (2) with loss of follow-up evaluations, (3) with a previous modified Rankin Scale of >1 and (4) without at least 2 results of blood glucose between the first 24 hours after admission. This study was approved by the Institutional Review Board of Chonnam National University Hospital and has therefore been performed in accordance with the ethical standards laid down in the 1964 Declaration of Helsinki and its later amendments. Written informed consent was not obtained due to the retrospective design of this study; therefore, the IRB of the hospital waived the need for written informed consent from participants.

### 2. Imaging protocol

We previously described our emergency imaging protocol for patients with acute ischemic stroke [14]. Briefly, patients underwent NCCT at the Emergency Department immediately after admission and underwent repeated imaging studies by MRI immediately after IV-tPA administration. The MRI protocol consisted of DWI, fluid-attenuated inversion recovery (FLAIR), gradient echo (GRE) imaging, time of flight MR angiography (MRA) and perfusion-weighted imaging (PWI) in sequence. Follow-up DWI/GRE was performed 24 hours after symptom onset. Additional follow-up imaging was performed 3–4 days after symptom onset. DWI/GRE (or sometimes brain CT) was performed if neurologic deterioration occurred.

### 3. Measurement of blood glucose

Blood glucose was measured by the Accu Check® test. It was measured immediately upon arrival in ER and after IV-tPA administration. Although this was a retrospective study, in our acute stroke protocol, blood glucose was measured every 6–8 hours (at least 4–6 times) during the first 24 hours after admission. Hyperglycemia was defined as a glucose level >180 mg/dL and managed by short-acting insulin in cases that exceeded 200 mg/dL according to our stroke protocol.

The various parameters of blood glucose were the following: blood glucose before IV-tPA administration (BG before IVT), blood glucose after IV-tPA administration (BG after IVT), mean blood glucose (mBG), maximal blood glucose (max BG), standard deviation of blood glucose (sdBG), and standard deviation of mean blood glucose (sdmBG).

### 4. Clinical assessment and outcome measurements

Baseline data collected from all patients included age, gender, NIHSS scores, and onset-to-treatment time. Stroke pathomechanisms were stratified according to the Trial of Org 10172 in Acute Stroke Treatment (TOAST) criteria after a complete diagnostic workup [15]. Hemorrhagic transformation (HT) was categorized as hemorrhagic infarction (type 1 or 2) or parenchymal hematoma (PH) (type 3 or 4) according to the guidelines established by a previous study [16]. Favorable outcomes were defined as mRS 0 to 2 at 90 days. Early mortality was defined as death within 90 days of symptom onset.

### 5. Statistical analysis

The percentage, mean (standard deviation, SD), or median (interquartile range, IQR) are reported depending on variable characteristics. Categorical variables were analyzed using the χ2-test and Fisher's exact test when appropriate. Continuous variables were analyzed using the independent samples *t*-test or the Mann–Whitney *U* test when appropriate. To evaluate which parameters of blood glucose were associated with early outcomes after IV-tPA administration, each parameter of blood glucose was adjusted by covariates of favorable outcomes at 3 months and death within 3 months (favorable outcomes; age, NIHSS, and atrial fibrillation, death; age, NIHSS, atrial fibrillation, and SBP). In addition, multiple logistic regression analysis was used to evaluate independent factors associated with PH; model 1 was adjusted by age, NIHSS score, hypertension, and atrial fibrillation; model 2 was adjusted by variables of model 1 and history of coronary artery disease. The associations between early outcomes and each parameter of the various blood glucoses levels were also analyzed by multiple logistic regression analysis. Adjustment was made for the variables of age, SBP, atrial fibrillation, NIHSS scores and white matter hyperintensities (WMH) for death at 3 months and age, NIHSS, atrial fibrillation and WMH for mRS 0–2 at 3 months. Odds ratios (ORs) and 95% confidence intervals (CIs) were calculated. A *p* value of <0.05 was considered to be statistically significant. All of the statistical analyses were performed using SPSS for Windows, version 17 (SPSS Inc., Chicago, IL, USA).

## Results

### 1. General characteristics

A total of 232 patients treated with IV-tPA were screened during the study period. Of these patients, 25 were excluded: 11 were excluded due to loss of follow-up, 8 were excluded due to incomplete work-up (such as lack of serial glucose evaluation or no imaging follow-up), 4 were excluded due to a previous mRS score of >1, and 2 were excluded due to Moyamoya disease. Ultimately, 207 patients (127 men and 80 women) were included in this study, and the mean age of the patients was 70.6±11.11 years. The general characteristics of the patients are summarized in Table 1. Seventy seven of 207 patients had favorable outcomes at 3 months. Patients with favorable outcomes were younger and had a higher frequency of atrial fibrillation, less severe WMH, lower baseline NIHSS scores, and lower levels of blood glucose parameters (BG after IVT, mBG, max BG and sdmBG). In addition, 33 patients had early mortality within 3 months. Patients with early mortality within 3 months were older and had a higher frequency of atrial fibrillation, coronary artery disease and severe WMH, higher baseline NIHSS scores and SBP, and higher levels of BG parameters (BG before/after IVT, mBG, max BG, sdBG and sdmBG).

**Table 1:** General characteristics of patients.

	mRS>2 (N = 130)	mRS 0–2 (N = 77)	p	Alive (N = 174)	Death (N = 33)	P
Age (mean±SD)	73.42±9.72	65.83±11.72	<0.001	69.37±10.92	77.03±9.94	<0.001
Male (n, %)	77 (59.2)	50 (64.9)	0.462	109 (62.6)	18 (54.5)	0.437
Risk factors						
HTN	88 (67.7)	45 (58.4)	0.230	107 (61.5)	26 (78.8)	0.074
DM	29 (22.3)	15 (19.5)	0.726	35 (20.1)	9 (27.3)	0.359
Dyslipidemia	8 (6.2)	9 (11.7)	0.193	15 (8.6)	2 (6.1)	>0.999
AF	48 (36.9)	17 (22.1)	0.030	44 (25.3)	21 (63.6)	<0.001
CAD	12 (9.2)	5 (6.5)	0.605	11 (6.3)	6 (18.2)	0.035
Previous stroke	12 (9.2)	10 (13.0)	0.485	16 (9.2)	6 (18.2)	0.131
Smoking	45 (34.6)	35 (45.5)	0.141	72 (41.4)	8 (24.4)	0.079
NIHSS (med, IQR)	12.0 (7.0)	7.0 (4.50)	<0.001	9.0 (8.0)	13.0 (5.50)	0.003
Onset to IVT time (mean±SD)	129.91±55.38	130.54±50.70	0.935	128.70±52.66	137.75±58.35	0.375
TOAST			0.802			0.846
LAA	32 (24.6)	29 (37.7)		58 (33.3)	3 (9.1)	
CE	65 (50.0)	32 (29.9)		65 (37.4)	23 (69.7)	
SVO	2 (1.5)	2 (2.6)		4 (2.3)	0	
OE	1 (0.8)	1 (1.3)		2 (1.1)	0	
UD	30 (23.1)	22 (28.6)		45 (25.9)	7 (21.2)	
Initial SBP	140.23±22.08	141.31±27.14	0.755	138.43±24.00	152.12±20.88	0.003
BG parameters						
BG before IVT	134.73±40.47	130.12±47.93	0.473	130.48±42.71	145.81±44.53	0.063
BG after IVT	144.88±42.21	118.42±33.17	<0.001	129.84±37.07	162.75±49.88	<0.001
Mean BG	139.04±36.66	120.55±29.90	<0.001	12779±31.73	155.19±44.35	<0.001
Max BG	164.20±53.20	147.72±48.29	0.027	151.93±45.50	190.42±69.86	0.004
SD of BG	21.27±17.19	22.16±17.52	0.712	20.05±14.99	29.70±25.00	0.003
SD of mean BG	0.14±0.08	0.17±0.11	0.034	0.15±0.09	0.17±0.12	0.161
Severe WMH	46 (35.4)	15 (19.5)	0.018	46 (26.4)	15 (45.5)	0.037
Microbleeds	14 (10.8)	9 (11.7)	0.823	19 (10.9)	4 (12.1)	0.768

HTN, hypertension; DM, diabetes mellitus; AF, atrial fibrillation; CAD, coronary artery disease; NIHSS, National Institutes of Health Stroke Scale; IVT, intra-venous thrombolysis; SD, standard deviation; TOAST, Trials of Organization; BP, blood pressure; BG, blood glucose; SD, standard deviation; WMH, white matter hyperintensity.

### 2. Variable parameters of blood glucose and clinical outcomes

Blood glucose was measured at 2 intervals for 24 patients (11.4%), 3 intervals for 37 patients (17.9%), 4 intervals for 86 patients (41.5%), and 5 intervals or more for 60 patients (29.0%). The association between blood glucose parameters and clinical outcome (mRS>2 at 3 months and death within 3 months) is summarized in Table 2. In multivariate logistic regression, BG after IVT (OR 1.021, 95% CI 1.010–1.032, p<0.001), mBG (OR 1.019, 95% CI 1.007–1.032, p = 0.0.02), and max BG (OR 1.007, 95% CI 1.000–1.014, p = 0.045) were independently associated with mRS>2 at 3 months (model 1; adjusted by age, NIHSS, and atrial fibrillation). With a level of blood glucose >180 mg/dL, BG after IVT was the only parameter among these associated with mRS>2 at 3 months, with OR 7.341 (95% CI 1.800–29.935). Several parameters of blood glucose were also independently associated with early mortality within 3 months (BG after IVT: OR 1.014, 95% CI 1.004–1.025, p = 0.007; mBG: OR 1.016, 95% CI 1.004–1.029, p = 0.010; max BG: OR 1.011, 95% CI 1.002–1.020, p = 0.017, in model 2) (Table 2). While there was no statistically significant association between sdBG and mortality, there was a positive trend (Model 1: OR 1.026, 95% CI 1.002–1.051, p = 0.036; model 2: OR 1.024, 95% CI 0.998–1.050, p = 0.067).

**Table 2:** Variable parameters of blood glucose and clinical outcomes at 3 months.

	mRS>2 at 3month				mRS>2 at 3month			
	Model 1	p	Model 2	P	Model 1	p	Model 2	p
BG before IVT	1.003 (0.995–1.001)	0.425	1.003 (0.996–1.011)	0.392	1.007 (0.998–1.016)	0.143	1.006 (0.997–1.016)	0.190
>180 mg/dL	0.962 (0.339–2.731)	0.942	1.032 (0.360–2.960)	0.953	3.131 (1.036–9.463)	0.043	2.901 (0.921–9.139)	0.069
BG after IVT	1.021 (1.010–1.032)	<0.001	1.021 (1.010–1.032)	<0.001	1.015 (1.005–1.025)	0.002	1.014 (1.004–1.025)	0.007
>180 mg/dL	7.341 (1.800–29.935)	0.005	7.133 (1.733–29.366)	0.007	3.128 (1.095–8.935)	0.033	2.629 (0.865–7.988)	0.088
Mean BG	1.019 (1.007–1.032)	0.002	1.019 (1.007–1.032)	0.002	1.017 (1.005–1.030)	0.006	1.016 (1.004–1.029)	0.010
>180 mg/dL	3.409 (0.860–13.514)	0.081	3.443 (0.864–13.714)	0.080	3.987 (1.312–12.119)	0.015	3.391 (1.076–10.684)	0.037
Maximal BG	1.007 (1.000–1.014)	0.045	1.007 (1.000–1.014)	0.047	1.012 (1.003–1.020)	0.008	1.011 (1.002–1.020)	0.017
>180 mg/dL	1.877 (0.831–4.237)	0.130	1.900 (0.839–4.303)	0.124	3.414 (1.376–8.467)	0.008	3.334 (1.292–8.603)	0.013
SD of BG	0.993 (0.974–1.013)	0.500	0.993 (0.974–1.013)	0.481	1.026 (1.002–1.051)	0.036	1.024 (0.998–1.050)	0.067
SD of mean BG	0.025 (0.001–0.804)	0.037	0.022 (0.001–0.716)	0.032	6.874 (0.135–350.653)	0.337	11.739 (0.106–1299.868)	0.305

mRS>2 at 3 month: Model 1 was adjusted by age, NIHSS, and AF. Model 2 was adjusted by variables with p<0.1 (age, AF, NIHSS, and severe WMH).

Death at 3 month: Model 1 was adjusted by age, NIHSS, AF, and SBP; Model 2 was adjusted by variables with P<0.1 (HTN, CAD, smoking, AF, WMH, and SBP).

In addition, we analyzed the relationship between various parameters of blood glucose and clinical outcomes according to the levels of blood glucose; <100 mg/dL, 100–180 mg/dL, and >180 mg/dL (Table 3). All parameters of blood glucose over 180 mg/dL were associated with a higher frequency of death within 3 months than those less than 180 mg/dL. BG after IVT, mBG and max BG over 180 mg/dL had a lower frequency of favorable outcomes at 3 months than those less than 180 mg/dL. After adjustment by covariates, BG after IVT and mBG over 180 mg/dL were independently associated with early mortality within 3 months and negatively associated with favorable outcomes at 3 months (Table 2). Also, max BG over 180 mg/dL was associated with early mortality within 3 months (Table 2).

**Table 3:** Various outcomes according to the level of blood glucose.

	<100 mg/dL	100–180 mg/dL	>180 mg/dL	P
BG before IVT	(n = 35)	(n = 138)	(n = 24)	
Parenchymal hemorrhage (n, %)	2 (5.7)	20 (14.5)	4 (16.7)	0.183
mRS 0–2 at 3 month	18 (51.4)	46 (33.3)	8 (33.3)	0.105
Death at 3 months	3 (8.6)	21 (15.2)	9 (37.5)	0.006
BG after IVT	(n = 35)	(n = 143)	(n = 28)	
Parenchymal hemorrhage (n, %)	1 (2.9)	19 (13.3)	7 (25.0)	0.010
mRS 0–2 at 3 month	24 (68.6)	49 (34.3)	3 (10.7)	<0.001
Death at 3 months	1 (2.9)	21 (14.7)	11 (39.3)	<0.001
Mean BG	(n = 31)	(n = 153)	(n = 23)	
Parenchymal hemorrhage (n, %)	1 (3.2)	18 (11.8)	8 (34.8)	0.001
mRS 0–2 at 3 month	20 (64.5)	54 (35.3)	3 (13.0)	<0.001
Death at 3 months	1 (3.2)	22 (14.4)	10 (43.5)	<0.001
Maximal BG	(n = 10)	(n = 147)	(n = 50)	
Parenchymal hemorrhage (n, %)	1 (10.0)	17 (11.6)	9 (18.0)	0.254
mRS 0–2 at 3 month	7 (70.0)	56 (38.1)	14 (28.0)	0.024
Death at 3 months	0	17 (11.6)	16 (32.0)	<0.001

### 3. Associations between parenchymal hematoma and blood glucose

Our data also analyzed the association between parameters of blood glucose and PH (Tables 4 and 5). Eighty five patients had HT and 27 had PH. Patients with PH were older and had a higher frequency of hypertension, atrial fibrillation, dyslipidemia and coronary artery disease than those without PH. In addition, those with PH had higher baseline NIHSS scores and higher levels of parameters of blood glucose (BG after IVT, mBG, and max BG) than those without PH. Among the various parameters, BG after IVT was independently associated with PH, adjusted by age, NIHSS, HTN, and AF, not including CAD(model 1; OR 1.010, 95% CI 1.001–1.019, p = 0.035: model 2; OR 1.009, 95% CI 1.000–1.018, p = 0.059). Also, mBG>180 mg/dL was associated with early mortality, adjusted by age, NIHSS, HTN, and AF, not including CAD (model 1; OR 2.897, 95% CI 1.016–8.259, p = 0.047: model 2; OR 2.618, 95% CI 0.897–7.638, p = 0.078).

**Table 4:** Characteristics of variables in patients with parenchymal hemorrhage and hemorrhagic transformation.

	No PH (N = 180)	PH (N = 27)	p	No HT (N = 122)	HT (N = 85)	P
Age (mean±SD)	70.2±11.6	73.2±7.0	0.065	69.8±11.3	71.7±10.8	0.218
Male (n, %)	109 (60.6)	18 (66.7)	0.673	69 (56.6)	58 (68.2)	0.111
Risk factors						
HTN	110 (61.1)	23 (85.2)	0.017	77 (63.1)	56 (65.9)	0.768
DM	36 (20.0)	8 (29.6)	0.311	21 (17.2)	23 (27.1)	0.120
Dyslipidemia	12 (6.7)	5 (18.5)	0.053	9 (7.4)	8 (9.4)	0.616
AF	51 (28.3)	14 (51.9)	0.024	27 (22.1)	38 (44.7)	0.001
CAD	12 (6.7)	5 (18.5)	0.053	8 (6.6)	9 (10.6)	0.315
Previous stroke	18 (10.0)	4 (14.8)	0.500	14 (11.5)	8 (9.4)	0.819
Smoking	71 (39.4)	9 (33.3)	0.673	49 (40.2)	31 (36.5)	0.664
NIHSS (med, IQR)	9.5 (7.0)	12.0 (6.0)	0.030	9.0 (7.0)	12.0 (7.0)	0.011
Onset to IVT time (mean±SD)	131.3±53.0	122.4±57.4	0.426	131.0±50.7	128.9±57.7	0.785
Initial SBP	140.33±24.49	142.59±20.86	0.650	142.1±25.5	138.6±21.8	0.307
BG parameters						
BG before IVT	131.58±43.8	142.69±39.1	0.193	129.5±37.0	138.4±51.3	0.159
BG after IVT	131.35±37.2	160.11±55.7	0.015	128.4±35.6	144.7±46.4	0.007
Mean BG	129.39±33.6	150.65±42.0	0.017	126.9±32.5	139.8±38.0	0.009
Max BG	155.51±50.8	175.14±56.9	0.067	153.0±46.7	165.4±58.2	0.105
SD of BG	21.42±7.6	22.71±15.2	0.717	21.5±16.3	21.7±18.6	0.923
SD of mean BG	0.16±0.1	0.14±0.1	0.244	0.16±0.1	0.15±0.1	0.208
Severe WMH	55 (30.6)	6 (22.2)	0.498	41 (33.6)	20 (23.5)	0.125
Microbleeds	21 (11.7)	2 (7.4)	0.745	17 (13.9)	6 (7.1)	0.177

**Table 5:** The associations between parenchymal hemorrhage and variable parameters of blood glucose.

	Model 1	p	Model 2	P
BG before IVT	1.003 (0.994–1.012)	0.549	1.003 (0.959–1.051)	0.873
>180 mg/dL	0.970 (0.284–9.990)	0.962	0.959 (0.278–3.299)	0.946
BG after IVT	1.010 (1.001–1.019)	0.035	1.009 (1.000–1.018)	0.059
>180 mg/dL	1.590 (0.553–4.569)	0.390	1.386 (0.466–4.125)	0.558
Mean BG	1.009 (0.998–1.020)	0.096	1.009 (0.998–1.020)	0.115
>180 mg/dL	2.897 (1.016–8.259)	0.047	2.618 (0.897–7.638)	0.078
Maximal BG	1.003 (0.995–1.010)	0.466	1.002 (0.995–1.010)	0.555
>180 mg/dL	1.215 (0.478–3.090)	0.682	1.156 (0.447–2.991)	0.765
SD of BG	0.995 (0.972–1.019)	0.699	0.994 (0.970–1.017)	0.596
SD of mean BG	0.050 (0.000–6.904)	0.234	0.039 (0.000–5.631)	0.202

Model 1; adjusted by age, NIHSS, HTN, and AF.

Model 2; adjusted by age, NIHSS, HTN, AF, and CAD.

## Discussion

The results of our study showed that high levels of BG after IVT, mBG and max BG were independently associated with mRS>2 at 3 months and death within 3 months. Among the parameters exceeding 180 mg/dL of blood glucose, BG after IVT was independently associated with mRS>2 at 3 months and mBG and max BG with death within 3 months while sdBG showed a non-significant yet positive trend with death within 3months. These results suggest that a single measurement of BG levels after IVT could be better for predicting prognosis than admission blood glucose and that mBG, max BG, or sdBG may better predict death within 3 months than single BG before and after IVT. In other words, it is suggested that serial blood glucose exams rather than single BG exams before IVT can possibly be more useful in predicting outcome in acute ischemic stroke.

Our study presents different implications compared to previous studies. Most previous studies pointed to admission hyperglycemia as a predictor of poor outcome [2], [4], [17]. In contrast, our study found that BG before IVT (may be admission BG in other studies) could not predict early outcome. These differences in findings may be attributed to the influence of stroke severity on blood glucose levels and that changes in BG after IVT cannot be predicted by admission blood glucose. Moreover, while the previous studies focused on admission blood glucose, the time point of glucose measurement was not clear in some studies. Since stroke is highly dynamic in the early stages, this variability can also extend to blood glucose levels.

In our study, mBG was more associated with clinical outcome than single shot blood glucose before IVT. According to other studies, mean glucose during ICU admission is also related to mortality in critical illnesses like sepsis and trauma [12], [18]. This therefore gives weight to the clinical implications of the serial blood glucose measurements taken during the 24 hours after IVT in our study. Due to the advantage that initial blood glucose was measured before IVT and we observed the changes of blood glucose over time, our study could actually have greater reliability than other studies. Previous studies reported that findings of persistent hyperglycemia for re-measurements after around 24 hours could create differences in outcome [6], [19]. However, our study is different in that we investigated the clinical significance of a wide range of parameters throughout the 24-hour timeframe using serial blood glucose measurements such as mBG, max BG, sdBG, and sdmBG. This may give value to our study as it might be one of the few studies to investigate the clinical significance of such various parameters. While our study did not confirm persistent hyperglycemia, our finding that various glucose parameters over 180 mg/dL were associated with poor outcome supports the results of previous studies.

Ischemic penumbra is likely to be found in the early hours after stroke onset but may also be seen up to 24 hours [20]. Hyperglycemia is associated with an increased recruitment of ischemic penumbra into irreversible infarction and poor outcome, due to increase brain lactate production [21], [22]. Furthermore, in the IVT patients, hyperglycemia has been shown to hamper the fibrinolytic processes delaying reperfusion of the hypoperfused at-risk tissue. Therefore, it might not only be admission hyperglycemia that needs to be managed but also hyperglycemia during the following 24 hours that could require continuous management. The results of our study support this hypothesis. In addition, recent studies have indicated that glycemic variability has more deleterious effects on the development of vascular complications in patients with diabetes mellitus than sustained hyperglycemia because acute glucose fluctuation activate the oxidative stress and exaggerates inflammation [23], [24]. In our study, high values of sdBG were also associated with death within 3 months, though non-significant. More measurements of blood glucose may increase the importance of sdBG. Because of the retrospective nature of this study, the numbers of BG measurement was not consistent and 39% of patients were measured only 2 or 3 times during a span of 24 hours. Further study for the clinical implications of sdBG in AIS will be needed.

Although the investigators sought to evaluate the effect of glucose lowering through intensive treatment as attempted by several clinical studies, they were unable to verify the effect of intensive insulin therapy (IIT) [25]–[27]. It was suggested that this might be due to a methodological problem of IIT. One recent study found that insulin treatment could lead to an increase in infarct volume [27] In addition, recent meta-analysis showed that there was no difference between treatment groups and control groups in functional outcomes and death [28]. However, our study still shows that mBG, max BG and/or sdBG were associated with early outcome and death within 3 months. Therefore, even without verifying the effect of insulin treatment, the need for continuous glucose control can be supported. The lack of benefit from glucose management might be due to a methodological problem of IIT. Since insulin therapy employs either continuous insulin infusion or setting controlled glucose levels at low levels, there is an increased risk of hypoglycemia, thereby presenting methodological problems to these studies. As a consequence of the methodological problems involved with glucose control, there may be a need to consider other treatment methods besides insulin. Nonetheless, our study neither attempts to compare the effects of insulin nor glucose lowering but rather represents an investigation study that shows that glucose measurements might be helpful for predicting clinical outcomes. As a result, we are currently conducting prospective studies on serial glucose monitoring and glucose control.

Importantly, the results of our study reveal the clinical significance associated with early max BG. Max BG was indeed associated with death at 3 months and mRS>2. Fuentes et al showed that hyperglycemia ≥155 mg/dL at any time within the first 48 hours from stroke onset, is associated with poor outcome [29]. Similarly, our study demonstrated that it was important to lower blood glucose levels that exceeded 180 mg/dL that were measured by serial blood glucose exams. This may provide evidence for continuous glucose monitoring as a means of glucose management.

In our study, there was furthermore a higher likelihood of future occurrence of PH in patients with higher BG after IVT. On the other hand, BG before IVT was not associated with PH, where mBG>180 mg/dL was significantly associated with PH. These results are different from previous studies that reported that BG before IVT could predict PH. Therefore, our study suggests the need for blood glucose lowering even if it is after IVT.

There are several limitations in this study. First, it has the inherent limitations of a retrospective observational study in a single center with a modest sample size. Although this was a retrospective study, we prospectively collected data in consecutive patients. Second, recanalization, which could affect prognosis, was not analyzed in this study. Because we performed NCCT-based IVT, we could not confirm recanalization in the patients. Previously, in models with reperfusion, hyperglycemia increased infarct size, while in animals without reperfusion, hyperglycemia seemed to have no adverse effect and might even have been beneficial [30], [31]. However, insulin had a non-significant and significantly heterogeneous effect according to the various models [32]. In addition, because of the retrospective nature of this study, we did not collect data on insulin treatment. Insulin may have a confounding effect on stroke outcome and poststroke hyperglycemia. Future prospective studies will have to take this confounding effect into account. Still, there is a limitation in that besides insulin treatment, there is not yet any other suitable treatment, making it difficult to judge which method will be best. Finally, there was a limitation in that blood glucose measurement times and counts were irregular. In cases in which glucose measurements were frequent, there was a tendency for higher blood glucose levels and thus a likelihood for worse outcome. However, even in cases in which blood glucose levels were normal, frequent measurements would likely yield similar results and therefore it can be assumed that this would not affect our results. In order to confirm our results, further prospective study would be needed.

In conclusion, serial measurements of blood glucose might be a better predictor of clinical outcome in patients with acute ischemic stroke treated with IVT than single blood glucose measurements. Among the variable parameters, BG before IVT was not associated with clinical outcome in this study. Therefore, these results suggest that variable parameters of blood glucose could be important for the prediction of clinical outcome in acute ischemic stroke treated with IVT.
