# Correction: Various Blood Glucose Parameters that Indicate Hyperglycemia after Intravenous Thrombolysis in Acute Ischemic Stroke Could Predict Worse Outcome

**DOI:** 10.1371/journal.pone.0105128

**Published:** 2014-08-15

**Authors:** The PLOS ONE Staff

There are errors in the equal contributions designation. The first and second authors, Deok-Sang Yoo and Jane Chang, should be noted as having contributed equally to this work.
